# The Estimation and Projection Package Age-Sex Model and the r-hybrid model: new tools for estimating HIV incidence trends in sub-Saharan Africa

**DOI:** 10.1097/QAD.0000000000002437

**Published:** 2019-11-12

**Authors:** Jeffrey W. Eaton, Tim Brown, Robert Puckett, Robert Glaubius, Kennedy Mutai, Le Bao, Joshua A. Salomon, John Stover, Mary Mahy, Timothy B. Hallett

**Affiliations:** a MRC Centre for Global Infectious Disease Analysis, Department of Infectious Disease Epidemiology, Imperial College London, London, UK; b Research Program, East-West Center, Honolulu, Hawaii; c Avenir Health, Glastonbury, Connecticut, USA; d National AIDS Control Council, Nairobi, Kenya; e Department of Statistics, Pennsylvania State University, University Park, Pennsylvania; f Department of Medicine, Stanford University School of Medicine, Stanford, USA; g Joint United Nations Programme on HIV/AIDS (UNAIDS), Geneva, Switzerland.

**Keywords:** antenatal clinic surveillance, HIV incidence, HIV prevalence, mathematical model, national household survey, sub-Saharan Africa

## Abstract

**Objectives:**

Improve models for estimating HIV epidemic trends in sub-Saharan Africa (SSA).

**Design:**

Mathematical epidemic model fit to national HIV survey and ANC sentinel surveillance (ANC-SS) data.

**Methods:**

We modified EPP to incorporate age and sex stratification (EPP-ASM) to more accurately capture the shifting demographics of maturing HIV epidemics. Secondly, we developed a new functional form for the HIV transmission rate, termed ‘r-hybrid’, which combines a four-parameter logistic function for the initial epidemic growth, peak, and decline followed by a first-order random walk for recent trends after epidemic stabilization. We fitted the r-hybrid model along with previously developed r-spline and r-trend models to HIV prevalence data from household surveys and ANC-SS in 177 regions in 34 SSA countries. We used leave-one-out cross validation with household survey HIV prevalence to compare model predictions.

**Results:**

The r-hybrid and r-spline models typically provided similar HIV prevalence trends, but sometimes qualitatively different assessments of recent incidence trends because of different structural assumptions about the HIV transmission rate. The r-hybrid model had the lowest average continuous ranked probability score, indicating the best model predictions. Coverage of 95% posterior predictive intervals was 91.5% for the r-hybrid model, versus 87.2 and 85.5% for r-spline and r-trend, respectively.

**Conclusion:**

The EPP-ASM and r-hybrid models improve consistency of EPP and Spectrum, improve the epidemiological assumptions underpinning recent HIV incidence estimates, and improve estimates and short-term projections of HIV prevalence trends. Countries that use general population survey and ANC-SS data to estimate HIV epidemic trends should consider using these tools.

## Abstract 2

Supplemental Digital Content is available in the text

## Introduction

In sub-Saharan Africa (SSA), key HIV epidemic indicators are estimated by fitting mathematical models to HIV prevalence data from sentinel surveillance among pregnant women attending antenatal care (ANC-SS) and nationally representative household surveys of the general adult population. Mathematical models combine epidemiologic information about natural history of HIV infection, population data, and the effects of antiretroviral treatment (ART) programmes to infer HIV incidence and AIDS mortality consistent with the observed HIV prevalence trends.

The *Estimation and Projection Package* (EPP) is a basic HIV epidemic model implemented within the Spectrum software for this purpose. EPP has developed incrementally, responding to evolving epidemic dynamics and context, data available from which to characterize the epidemic and demands on HIV estimates for policy and program purposes. EPP was initially conceived as a simple four-parameter HIV epidemic model capturing the HIV epidemic growth rate, start time, epidemic peak, and stabilization following initial decline [1,2]. Parameter inference from ANC-SS HIV prevalence was initially via maximum likelihood [3] and subsequently probabilistic Bayesian inference with hierarchical random-effects for ANC sentinel sites [4]. Reflecting accumulation of more heterogeneous HIV prevalence trajectories following the initial epidemic peak and decline, further developments of EPP focused on using semiparametric functions to more flexibly represent changes in the HIV transmission rate over the course of the epidemic [5–10]. The model has been updated continuously to capture ART scale-up, changes in eligibility, and its effects for HIV survival and transmission [10–13].

Since 2013, the UNAIDS Reference Group has recommended the ‘r-spline’ model variant for most countries with multiple years of ANC-SS and national household surveys [10]. This model uses penalized B-splines (‘p-spline’) with seven basis functions to flexibly model the transmission rate *r* (*t*) over the course of the epidemic [7,8]. The model further imposes an ‘equilibrium prior’ assumption that, beyond the end of data observation, the transmission rate will be drawn towards a value guided by the transmission rate required to maintain an equilibrium prevalence at current level in the absence of any effects of ART on survival or HIV transmission [8]. Beyond the last observed data point, the spline function is truncated and replaced by a first-order random walk on the log-scale [8]. The priority guiding this model specification was to ensure stable and reliable estimates and short-term projections for HIV prevalence from relatively sparse ANC-SS and national HIV survey data, for example, for estimating treatment need and coverage. However, as noted by Hogan and Salomon [8], the ‘equilibrium’ assumption may now seem incongruous for estimating HIV incidence trends in an era in which HIV policy is intensely focused on rapidly reducing new HIV infections and ART is anticipated to be substantially affecting both survival and HIV transmission [14].

The other predominant recent transition has been a dramatic shift in the age profile of the epidemic as people living with HIV (PLHIV) survive to older ages with the scale-up of ART and incidence reductions resulting in lower prevalence amongst young adults than experienced by previous cohorts. This creates distinct trends in HIV prevalence observed among pregnant women compared with the general population [15], and a large and steadily increasing proportion of PLHIV on ART above age 50 years [10]. Adjustments have been incorporated to account for these dynamics in EPP [10,13], which considers the age 15–49-year population as a single homogenous group. However, an estimation framework is needed that endogenously captures the shifting demographics of the epidemic and explicitly simulates HIV prevalence among pregnant women accounting for patterns of age-specific fertility, HIV incidence and disease progression, and effects of HIV on fertility [16–18].

This article introduces two major updates to EPP 2018 and EPP 2019: the *EPP Age-Sex Model* (EPP-ASM) to explicitly capture the demographic dynamics of the HIV epidemic and the *r-hybrid* model, a new model for inferring the HIV transmission rate. We also describe small updates to the likelihood for directly observed HIV incidence trends in population surveys.

## Methods

### The Estimation and Projection Package Age-Sex Model

The EPP Age-Sex model (EPP-ASM) is a new framework that integrates the cohort-component demographic projection model of Spectrum with the basic infectious disease transmission dynamics of the EPP model, and the already harmonized representation of HIV natural history and impacts of ART programmes. The model represents the adult population aged 15 years and older by sex, single year of age, and HIV status, and mirrors the model structure and assumptions of the Spectrum model [19]. Technical details of the EPP-ASM model specification are described in Supplementary Information Section S1.

Similar to previous EPP model formulations, the HIV incidence rate *λ*_15–49_(*t*) at time *t* is determined by the transmission rate *r*(*t*) among untreated HIV-positive adults, the HIV prevalence *ρ*_15–49_(*t*) among adults aged 15–49 years, the proportion of HIV-positive adults on ART *α*_15–49_(*t*), and the average percentage reduction in HIV transmission per percentage increase in ART coverage (*ω*): 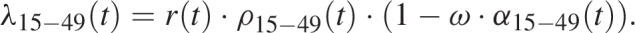


The r-spline, r-trend and the new r-hybrid EPP model variants for *r*(*t*) [13] can be used within the EPP-ASM framework. The default value for *ω* is 0.7. Although population surveys consistently find that viral suppression amongst persons on ART is between 85 and 95% and studies conclusively demonstrate that persons who are virally suppressed do not transmit HIV [20], the lower value of *ω* = 0.7 reflects that the average population impact of an increase in ART coverage is somewhat less. This because people on ART are likely to be somewhat older, infected for longer durations, and on average have lower behavioral risks for onward transmission, attenuating the expected impact of increasing ART coverage on reducing incidence.

### r-hybrid model

The ‘r-hybrid’ model is a new functional form introduced in EPP 2019 for modelling the transmission rate *r*(*t*) among untreated adults over the course of the epidemic. The motivation for the r-hybrid model, and specifically the ‘hybrid’ moniker, was to combine one model for the initial stages of the epidemic and another for more recent trends. During the initial period of the epidemic, surveillance data were relatively sparse, often a handful of ANC sentinel sites, but epidemics followed relatively consistent pattern of exponential growth, peak, and decline. The r-hybrid model uses a four-parameter logistic function for these processes from the start of the epidemic in the 1970s through the mid-2000s: 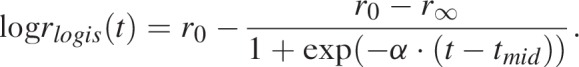


The four parameters of the logistic function provide structure to key stages of the epidemic. The first parameter *r*_0_ is the log of the initial epidemic growth rate, *r*_∞_ is the log endemic transmission rate after the epidemic has stabilized, *α* > 0 is the rate of decline of the log transmission rate as the epidemic saturates and *t*_*mid*_ is the inflection point of the logistic function. We specified diffuse prior distributions for these parameters as normal densities parameterized via the mean and standard deviation: 
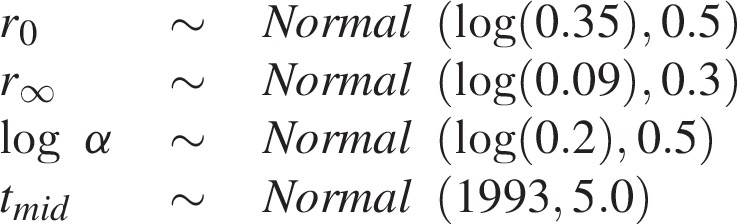


The prior mean for the log growth rate *r*_0_ corresponds to an initial epidemic doubling time of 2 years with 95% of prior mass between 0.7 and 5.3 years [21] and the prior mean for *r*_∞_ implies an endemic transmission rate of 0.09 per year with 95% of mass between 0.05 and 0.16.

From the mid-2000s, when HIV epidemics had stabilized to a state of endemic transmission dynamics, we used a piecewise-linear spline with a first-order random-walk (RW1) penalty on the spline coefficients to model changes in log *r*(*t*). The piecewise-linear spline allows the transmission rate to vary with recent epidemic trends, whereas the RW1 penalty imposes the assumption that the expected transmission rate remains steady with linearly increasing variance, rather than being drawn towards a particular value. Under the r-spline model, a RW1 process for log *r*(*t*) has been used for short-term epidemic projections past the last data point [9]. Using the RW1 process for modelling recent trends harmonizes the epidemiologic assumptions underpinning inference about recent trends with the assumptions for short-term projections. Supplementary Information Section S2 describes technical details of the random walk component.

### Data and likelihood

The statistical model for inference with the EPP-ASM and r-hybrid models are the same as for other existing EPP model variants. EPP utilizes two data sources for estimating model parameters and inferring epidemic trends. The first is HIV prevalence, and wherever available, incidence among the general population aged 15–49 years measured in nationally representative household surveys [10,13,22]. The second is HIV prevalence among pregnant women, measured through ANC sentinel surveillance [3] or routine HIV testing of pregnant women attending ANC [23]. Technical details of the likelihood are described Supplementary Information Section S3.

### Analysis

We applied the EPP-ASM model to data from 34 SSA countries that used EPP to create national HIV estimates submitted to UNAIDS in 2018 (Table 1). Countries stratify EPP estimation to subnational units reflective of differences in local epidemiology; we used these stratifications as defined by country estimate teams constituting a total of 177 EPP regions. We excluded countries that used key-population as stratifications (Cape Verde, Comoros, Madagascar, Mauritania, Mauritius, Niger, Senegal), with fewer than two household surveys with reliable HIV serological testing (Nigeria, South Sudan), and that did not use EPP to estimate HIV incidence trends (South Africa). We used data from Spectrum estimates files released by countries through UNAIDS 2018 HIV estimates with the following exceptions: we omitted household survey prevalence data from the Uganda 2011 AIS because of uncertainty about accuracy of HIV serological testing [24]; substituted prevalence estimates for the Zambia 2013–2014 DHS with adjusted estimates accounting for imperfect immunoassay performance through a Bayesian analysis [24]; reanalyzed Tanzania AIS survey and Zambia DHS survey prevalence according to current regional boundaries used in EPP; removed ANC-RT census prevalence from Namibia regions because inputted data were not calculated at the regional level; and removed artificially constructed ‘ANC pseudo-site’ prevalence inputs [25]. Fertility rate ratio parameters determining the prevalence of HIV among pregnant women were updated to default parameter values in Spectrum 2019.

**Table 1:** Summary of data used for *Estimation and Projection Package* model analysis.

UN region	Country	EPP regions^a^	HH surveys w/ HIV^b^	ANC-SS sites	ANC-SS observations	Site-level ANC-RT observations	Years ANC-RT census prevalence	Last data year
Eastern	Burundi	2	4	24	98	57	4	2017
	Eritrea	2	1	16	122			2017
	Ethiopia	18	3	123	746	376		2017
	Kenya	8	4	40	518	192	5	2017
	Malawi	3	4	54	249	378	7	2017
	Mozambique	11	2	39	263	131	4	2017
	Rwanda	2	3	30	169	237	3	2017
	Uganda	2	2	43	430	249	6	2017
	United Rep. Tanzania	27	4	199	946	484		2017
	Zambia	10	4	24	168	144	6	2017
	Zimbabwe	10	5	68	250	313	1	2017
Southern	Botswana	2	3	24	244		4	2017
	Lesotho	2	4	17	108	35	3	2017
	Namibia	14	2	40	329	187		2017
	Swaziland	4	3	21	100			2016
Middle	Angola	2	1	48	192			2016
	Cameroon	2	2	79	315	231		2017
	Central African Republic	2	2	42	118			2015
	Chad	2	1	34	97	34		2017
	Congo	2	2^c^	47	110	6	5	2017
	Dem. Rep. Congo	2	2	67	360			2015
	Equatorial Guinea	1	3	2	14		5	2017
	Gabon	2	1	27	62	57	2	2017
Western	Benin	12	2	60	886	333	6	2017
	Burkina Faso	2	2	13	233	84	6	2017
	Côte d’Ivoire	11	2	74	209			2017
	Gambia	2	1	12	120			2017
	Ghana	2	2	40	756	12	1	2017
	Guinea	2	2	32	101			2015
	Guinea-Bissau	2	1	18	69	138	8	2017
	Liberia	2	2	33	126			2017
	Mali	2	3	31	103	16	4	2017
	Sierra Leone	2	4	13	54			2013
	Togo	6	2	90	521	97	3	2017
Eastern	(11 countries)	95 [93]	36 [35]	660	3959	2561	36	
Southern	(4 countries)	22 [22]	12 [12]	102	781	222	7	
Middle	(8 countries)	15 [8]	14 [11]	346	1268	382	12	
Western	(11 countries)	45 [35]	23 [19]	416	3178	878	28	
Total	(34 countries)	177 [158]	85 [77]	1524	9186	443	83	

ANC-RT, antenatal care routine HIV testing; ANC-SS, antenatal clinic sentinel surveillance; EPP, *Estimation and Projection Package*; HH, household.

^a^Countries with two EPP regions typically stratify EPP estimation by Urban/Rural regions. Countries with greater than two are typically stratified by first-level administrative units. Number of EPP regions in brackets at the table bottom indicate number of EPP regions represented in leave-one-out cross validation exercise.

^b^The number of household surveys with HIV prevalence observations used in model fitting. Data were used as entered into EPP by countries in the 2018, with the following exceptions: Uganda 2011 AIS survey was removed; estimates from Zambia 2013–2014 DHS were updated based on results of a Bayesian analysis to account for imperfect assay performance; Zambia 2002 and 2007 DHS and all Tanzania AIS were re-analysed to reflect current administrative boundaries. Countries with more than one survey were included in leave-one-out cross validation exercise. Number in brackets at the table bottom indicate number of household surveys represented in leave-one-out cross validation exercise.

^c^The first Congo survey was Urban only, therefore only ‘Congo – Urban’ is used in the leave-one-out cross validation exercise.

We fitted the r-hybrid, r-spline, and r-trend versions of the EPP-ASM model to each of the 177 EPP-ASM regions. We used the Incremental Mixture Importance Sampling (IMIS) algorithm to approximately sample from the posterior distribution [26]. For the IMIS algorithm, we used 100 000 initial samples for the r-spline model and 10 000 for the r-trend and r-hybrid models, 1000 samples at each IMIS iteration, optimization steps every five iterations up to the 25th iteration, and retained 3000 resamples from the joint posterior distribution (see [26] for details of the IMIS algorithm).

For model validation and comparison, we conducted leave-one-out cross-validation using national survey prevalence estimates. For 158 EPP regions which had two or more national surveys with HIV prevalence, we refitted each of the three models (r-hybrid, r-spline, r-trend) withholding a single national survey prevalence data point and including all other survey and ANC prevalence data, a total of 470 fits for each model. We generated samples from the posterior predictive distribution for the withheld prevalence data point on the probit scale by sampling a single value for each sample from the posterior distribution from a normal distribution with mean given by the model predicted prevalence in the given survey year and standard deviation 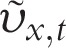.

We used the continuous ranked probability score (CRPS) and the expected log predictive density (ELPD) to compare predictive performance of the three models. CRPS is a measure of model prediction error analogous to mean absolute error (MAE) suitable for probabilistic forecasts [27]. Smaller values indicate a smaller forecasting error and hence better prediction. CRPS was calculated on the percentage-point scale using the sample CRPS approximation implemented in the R *scoringRules* package [28]. ELPD is approximated by calculating the average likelihood over all posterior samples for each withheld data point, taking the log and summing; higher values indicate a greater expected likelihood and hence better prediction [29].

R implementations of the EPP-ASM and r-hybrid models are available at http://github.com/mrc-ide/eppasm. Computer code for reproducing all analyses is available from: https://github.com/jeffeaton/eppasm-rhybrid-paper.

## Results

**Fig. 1:**
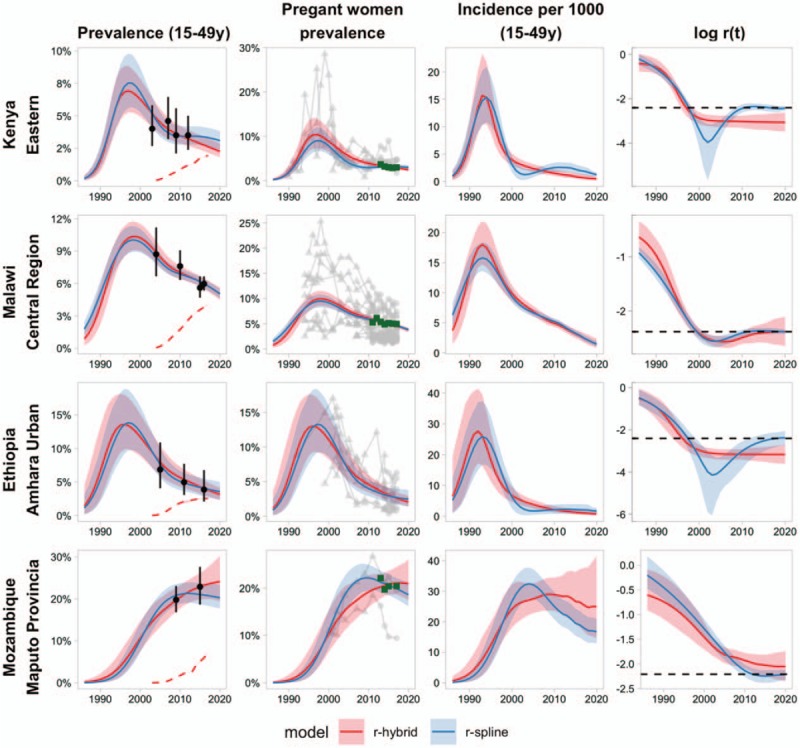
Examples of r-hybrid model fits (red) compared with r-spline (blue) model fitted using the EPP-ASM model for Kenya – Eastern (top), Malawi – Central Region, Ethiopia – Amhara Urban, and Mozambique – Maputo Province (bottom).

Figure 1 illustrates examples of model fits with the r-hybrid model using the EPP-ASM to four EPP regions chosen to illustrate different characteristic patterns of how the transmission rate affects epidemic estimates: Kenya – Eastern, Malawi – Central Region, Ethiopia – Amhara Urban, and Mozambique – Maputo Province. Across all regions, the transmission rate *r*(*t*) is high during early stages of the epidemic when HIV prevalence and incidence rate are increasing exponentially, then declines in the 1990s as incidence peaks and declines. HIV prevalence peaks 4--6 years after incidence, except in Maputo Province where incidence was estimated to peak in 2009 and prevalence continues increasing.

Solid trend lines indicate posterior means and shaded areas 95% credible ranges estimates. Left panel: illustration of HIV prevalence among age 15–49 years. Points and vertical ranges indicate household survey prevalence estimates and 95% CIs. Red dashed lines indicate the proportion of the age 15–49 population on ART. Middle-left panel: illustrates HIV prevalence among pregnant women. Green squares are ANC-RT census HIV prevalence aggregated for all ANC attendees in the region. Each grey line is data from one ANC-SS site; triangles are sentinel survey observations and grey circles are observations from ANC-RT at SS sites. ANC-SS observations have been adjusted on the probit scale by the posterior mean for the ANC-SS bias parameter. Middle-right panel: illustration of the HIV incidence rate per 1000 person-years among age 15–49 years. Right panel: illustrates the log transmission rate (*r*(*t*)) among untreated adults. The dashed horizontal line indicates the value for the r-spline equilibrium prior, *r*_*eq*_ = 1/(11.5·(1–*prev*)), based on the posterior mean prevalence in the last year of data. ART, antiretroviral therapy; ANC-SS, antenatal care sentinel surveillance; ANC-RT, antenatal care routine HIV testing; CI, confidence interval;

Both the r-hybrid and the r-spline models produce visually similar fits to HIV prevalence from national surveys and amongst pregnant women. However, in some cases, the models can produce qualitatively different trends for HIV incidence, which can be understood through assumptions about the transmission rate *r*(*t*). For example, in the area labelled ‘Kenya – Eastern’, with the r-spline model, the transmission rate declines rapidly, followed by an increase to the assumed equilibrium prior value. This results in an increasing incidence rate from the mid-2000s, even while ART coverage is scaling up, followed by declining incidence in recent years. The r-hybrid model estimates a similar steep decline in log *r*(*t*), but the transmission rate stabilizes and remains relatively steady, resulting in steadily declining HIV incidence from the mid-2000s onward. Both models estimate similar prevalence at the last survey in 2012, but the lower incidence estimated by the r-hybrid model results in an estimate of steadily decreasing prevalence compared with the stable prevalence trend estimated by the r-spline model.

In contrast, for ‘Malawi – Central Region’, the r-hybrid and r-spline model posterior mean estimates are very similar. The random-walk component of the r-hybrid model also estimates an increase in the transmission rate to the anticipated equilibrium prior value. However, rather than being constrained to this value, the uncertainty range about *r*(*t*) steadily increases in recent years as prevalence data become less informative about incidence trends. The relative standard error for the incidence rate in 2017 is 60% larger with the r-hybrid model than the r-spline model.

The example of ‘Ethiopia – Amhara Urban’ is similar to Kenya – Eastern with the increase in *r*(*t*) to the equilibrium prior resulting in an apparently flat HIV incidence trend over the past decade whereas the r-hybrid model estimates a steady transmission rate and decreasing incidence as untreated prevalence decreases. Unlike Kenya – Eastern, recent HIV prevalence estimates are very similar for both models, guided by the recent population survey in 2015.

In a final example, in ‘Mozambique – Maputo Province’, the transmission rate is decreasing during the random-walk period, but is higher than what would have been assumed by the equilibrium prior value. Consequently, the HIV incidence is higher, estimated to have peaked later, and have much larger uncertainty than with the r-spline model where the transmission rate is drawn to a lower level. The HIV prevalence was steadily increasing over the past decade.

### Parameter estimates

**Fig. 2:**
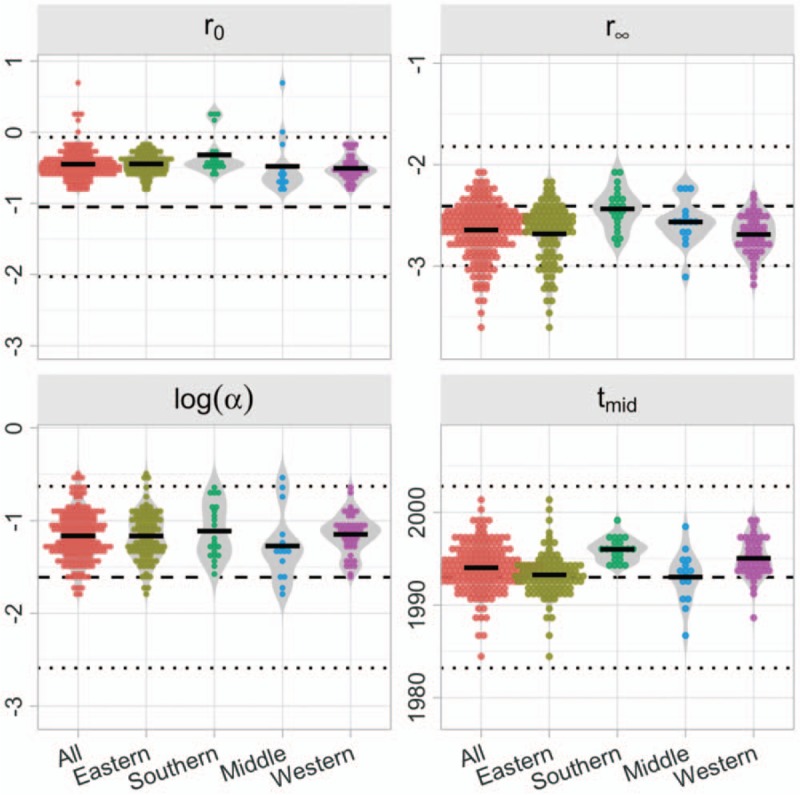
Posterior mean estimates of logistic function parameters for 177 *Estimation and Projection Package* regions.

Figure 2 illustrates estimates of the posterior means for the logistic function parameters of the r-hybrid model fitted to each of the 177 EPP regions relative to the prior distributions for each parameter. The average value for *r*_0_ was −0.45 with interquartile range (IQR) −0.57 to −0.36. The average *r*_∞_ was −2.64 (IQR −2.78 to −2.48). For log *α* the average was −1.16 (IQR −1.34 to −0.97). The average *t*_*mid*_ was 1994.0 with IQR 1992.7 to 1995.3. In the southern Africa region, the average value for the log initial epidemic growth rate *r*_0_ was −0.32, −2.44 for the log endemic transmission rate *r*_∞_, and 1996.0 for the inflection point *t*_*mid*_. The higher growth rate, higher endemic transmission rate, and later inflection are consistent with the larger epidemics and later emergence, peak, and decline of HIV epidemics in the southern Africa region compared to other regions.

Each dot reflects posterior mean from one EPP region. Solid black bars are average of posterior mean estimates for all EPP regions fit for all regions and by UN region country groupings. The dashed horizontal line reflects the prior mean for each parameter. Dotted horizontal lines reflect 2.5 and 97.5% quantiles of prior distribution for each parameter. EPP, *Estimation and Projection Package*.

### Model comparison and validation

Table 2 summarizes results of leave-one-out cross-validation for 470 household survey prevalence data points across 158 EPP regions with 2 or more household surveys. The r-hybrid model had the lowest CPRS and highest ELPD amongst the three models, indicating the best performance for out-of-sample prediction. Aggregating all regions, the difference in the CPRS and ELPD between the r-hybrid model and the r-spline or r-trend models was more than twice the standard error for the difference. The r-hybrid model featured better predictive interval coverage, with 95% predictive intervals containing the observed prevalence in 91.5% of cases compared with only 87.2 and 85.5% for the r-spline and r-trend models, respectively. Stratifying the comparison by UN country regions, the r-hybrid model had the lowest CRPS and highest ELPD in Eastern and Western Africa countries. The r-trend model had the lowest CRPS and highest ELPD in Middle and Southern regions, though the standard errors for the difference was large. The r-hybrid model had the highest predictive interval coverage in all regions.

**Table 2:** Results of leave-one-out cross-validation for 470 household survey prevalence data points in 158 *Estimation and Projection Package* regions.

UN region^a^	Model	CRPS^b^	CRPS difference (SE)^b^	ELPD^c^	ELPD difference (SE)^c^	80% interval coverage	95% interval coverage
All	r-hybrid	0.98	0.0	245.68	0.0	75.1%	91.5%
(470 fits)	r-spline	1.05	−0.07 (0.02)	171.52	74.2 (19.8)	70.4%	87.2%
	r-trend	1.07	−0.09 (0.03)	162.29	83.4 (19.2)	69.8%	85.5%
Eastern	r-hybrid	1.04	0.0	160.57	0.0	75.2%	91.3%
(323 fits)	r-spline	1.11	−0.07 (0.03)	123.74	36.8 (11.1)	72.1%	87.9%
	r-trend	1.16	−0.12 (0.04)	98.43	62.1 (14.8)	70.0%	86.4%
Middle	r-hybrid	0.66	0.0	7.5	0.0 (0.0)	70.6%	82.4%
(17 fits)	r-spline	0.72	−0.06 (0.09)	1.81	5.7 (4.2)	47.1%	82.4%
	r-trend	0.59	0.07 (0.07)	10.51	−3.0 (2.5)	70.6%	82.4%
Southern	r-hybrid	1.54	0.0	35.43	0.0	63.0%	85.2%
(54 fits)	r-spline	1.64	−0.09 (0.08)	25.31	10.1 (6.1)	51.9%	81.5%
	r-trend	1.47	0.07 (0.08)	40.04	−4.6 (5.9)	64.8%	79.6%
Western	r-hybrid	0.38	0.0	42.18	0.0	84.2%	98.7%
(76 fits)	r-spline	0.43	−0.05 (0.03)	20.66	21.5 (14.7)	81.6%	89.5%
	r-trend	0.52	−0.14 (0.04)	13.31	28.9 (10.2)	72.4%	86.8%

^a^Countries included in each region are reported in Table 1. All countries with more than one household survey with HIV are included (Eritrea, Angola, Chad, Gabon, Gambia, and Guinea-Bissau excluded).

^b^Continuous ranked probability score (CRPS) is measure of the average percentage-point prediction error comparing out-of-sample posterior predictive distributions for HIV prevalence among age 15–49 years to observed survey prevalence. Lower values indicate smaller predictive errors. Values in parentheses are estimates of the standard error (SE) for the difference in CRPS between the r-hybrid model and r-spline or r-trend models.

^c^Expected log predictive density (ELPD) is a measure for the expected log-likelihood for probit-transformed survey prevalence in leave-one-out cross validation. Higher values indicate more accurate predictions of withheld survey prevalence. Values in parentheses are the estimated standard error (SE) for the difference in ELPD between the r-hybrid model and r-spline or r-trend models.

Results were similar when restricting to evaluating out-of-sample predictions for the most recent prevalence survey (Table 3), indicative of model performance for short-term estimates and projections in the years following survey prevalence. The r-hybrid model had the lowest CRPS and the highest ELPD. The 95% predictive interval included the observed prevalence for 92.4% of cases for the r-hybrid model compared with 88.6 and 88.0% for the r-spline and r-trend models.

**Table 3:** Results of leave-one-out cross-validation for most recent household survey prevalence data points in 158 *Estimation and Projection Package* regions.

UN region^a^	Model	CRPS^b^	CRPS difference (SE)	ELPD^c^	ELPD difference (SE)	80% interval coverage	95% interval coverage
All	r-hybrid	0.98	0.0	80.08	0.0	81.6%	92.4%
(158 fits)	r-spline	1.06	0.08 (0.03)	40.31	−39.8 (16.6)	72.2%	88.6%
	r-trend	1.08	0.10 (0.04)	54.02	−26.1 (11.5)	72.2%	88.0%
Eastern	r-hybrid	1.09	0.0	46.91	0.0	80.6%	92.5%
(93 fits)	r-spline	1.17	0.08 (0.04)	33.68	−13.2 (6.4)	74.2%	90.3%
	r-trend	1.25	0.16 (0.06)	26.06	−20.9 (7.3)	69.9%	89.2%
Middle	r-hybrid	0.52	0.0	5.21	0.0	87.5%	87.5%
(8 fits)	r-spline	0.53	0.01 (0.08)	2.8	−2.4 (2.6)	50.0%	87.5%
	r-trend	0.47	−0.05 (0.09)	6.52	1.3 (1.9)	87.5%	87.5%
Southern	r-hybrid	1.66	0.0	7.85	0.0	72.7%	86.4%
(22 fits)	r-spline	1.83	0.17 (0.13)	0.18	−7.7 (5.2)	50.0%	77.3%
	r-trend	1.58	−0.08 (0.14)	11.37	3.5 (5.3)	63.6%	77.3%
Western	r-hybrid	0.35	0.0	20.12	0.0	88.6%	97.1%
(35 fits)	r-spline	0.41	0.06 (0.06)	3.65	−16.5 (14.3)	85.7%	91.4%
	r-trend	0.43	0.08 (0.04)	10.07	−10.0 (6.8)	80.0%	91.4%

SE, standard error.

^a^Countries included in each region are reported in Table 1. All countries with more than one household survey with HIV are included (Eritrea, Angola, Chad, Gabon, Gambia, and Guinea-Bissau excluded).

^b^Continuous ranked probability score (CRPS) is measure of the average percentage-point prediction error comparing out-of-sample posterior predictive distributions for HIV prevalence among age 15–49 years to observed survey prevalence. Lower values indicate smaller predictive errors. Values in parentheses are estimates of the standard error for the difference in CRPS between the r-hybrid model and r-spline or r-trend models.

^c^Expected log predictive density (ELPD) is a measure for the expected log-likelihood for probit-transformed survey prevalence in leave-one-out cross validation. Higher values indicate more accurate predictions of withheld survey prevalence. Values in parentheses are the estimated standard error for the difference in ELPD between the r-hybrid model and r-spline or r-trend models.

## Discussion

As the dynamics, programmatic response, and data about the HIV epidemic continue to evolve, the methods and tools with which the epidemic is characterized necessarily also continue to develop. We have described two major updates to the EPP model to improve estimates and short-term projections of HIV epidemics for countries in sub-Saharan Africa with general-population HIV epidemics. The EPP-ASM model, introduced in EPP 2017, captures the shifting demographics of the HIV epidemic as new infections reduce among young adults and PLHIV survive to older ages with availability of ART. The model endogenously reflects the relationship between HIV trends among pregnant women observed in ANC-based surveillance and population HIV trends.

The r-hybrid model, introduced in EPP 2019, is a new way to model the changes in the HIV transmission rate over the course of the epidemic, the fundamental quantity estimated by EPP. R-hybrid combines a simple parametric model to structure the growth, peak, and decline of the epidemic with a first-order random walk model for recent trends. The random-walk model allows data-driven flexibility in recent trends, while exerting the prior assumption of a steady transmission rate once the epidemic has stabilized. The r-hybrid model resulted in more regular patterns for recent HIV incidence trends, and demonstrated improved performance in out-of-sample prediction for HIV prevalence. Uncertainty ranges for HIV prevalence and incidence tended to be somewhat larger for the r-hybrid model, resulting in improved nominal coverage of posterior predictive intervals. We recommend that countries in sub-Saharan Africa with general population HIV surveillance consider the EPP-ASM and r-hybrid models for developing HIV epidemic estimates.

The EPP-ASM model improves the consistency of the EPP model with the Spectrum model, into which EPP estimates are inputted. Many features of the EPP-ASM model have long been present in the Thembisa model, an integrated demographic projection and HIV transmission-dynamic model for national HIV estimates, projections, and intervention prioritization in South Africa [30]. Important differences remain between the Thembisa approach and EPP-ASM, most notably the mechanistic representation of sexual mixing, transmission dynamics, and impacts of other interventions, such as condoms and medical male circumcision in Thembisa compared with the more phenomenological approach of semiparametrically modelling changes in the average transmission rate in EPP. Future research should investigate whether more mechanistic representation of HIV transmission in EPP can further improve estimates across other settings.

There are a number of limitations of the models. First, the models are not yet implemented for key population-stratified EPP estimation, which is a recommended practice for applying EPP in concentrated epidemic settings outside SSA and some low-level epidemics in SSA. This is a priority area for further development and will require specification of demographic structure for entry and exit from key population groups. Second, suboptimal and unknown data quality, particularly for historical sentinel surveillance and routine programmatic data, require incorporation of nonsampling error into statistical models and limit precise interpretations of observed small changes in prevalence time series [23,31]. Third, we were required to fix values for some key model parameters including the standard deviation of the random component of the r-hybrid model and the effect of increased ART coverage on transmission rate as data are insufficient to make inference about these parameters when fitting EPP independently to each region. Extensions to hierarchical model inference across subnational areas and countries and analysis of trends in population cohort data may improve characterization of these parameters in future.

However, the most substantial future improvements to estimates are likely to be derived from more granular modelling of existing data sources and incorporation of new data sources into estimates. The EPP-ASM model structure provides a foundation for this. Inference from age-stratified survey and sentinel surveillance prevalence data is a natural extension enabled by the existing model structure. Precise modelling of the impact of HIV on age-specific mortality will also facilitate inference about HIV epidemic trends from AIDS-specific and all-cause mortality data [32]. Mahiane *et al.* describe extension of the EPP-ASM model structure into the Spectrum CSAVR tool for inference from case surveillance and vital registration data in concentrated epidemic settings [33]. Similar data about HIV testing and diagnosis are now also routinely reported in sub-Saharan Africa. Maheu-Giroux and colleagues describe an extension of the EPP-ASM model structure to also estimate HIV testing rates from over time from survey and routine HIV testing programme data tracking progress toward the ‘first 90’ HIV knowledge of status targets [34]. Bringing this approach together with the EPP model to estimate recent HIV incidence trends from testing and diagnosis data is a logical next step for more granular and real-time tracking of epidemic trends and programme impacts.

## Acknowledgements

J.W.E., J.A.S., and T.B.H. conceived the work. J.W.E., T.B., R.G., L.B., J.S., M.M., and T.B.H. designed the work. J.W.E., T.B., and R.P. implemented the models. All authors critically reviewed model results throughout the model development process. All authors critically edited the manuscript for intellectual content.

Funding: This research was supported by UNAIDS, NIH R01-AI136664, and the Bill and Melinda Gates Foundation. We acknowledge joint MRC Centre funding from the UK Medical Research Council and Department for International Development via MRC MR/R015600/1.

We acknowledge Peter Young for substantial intellectual contribution to critically reviewing and critiquing model results, testing developmental model versions, and providing critical editing and comment on earlier versions of this manuscript.

We acknowledge the HIV Estimates Technical Teams from the 34 SSA countries who created and updated Spectrum files with national data sources utilized in this analysis. We thank participants of the UNAIDS Reference Group on Estimates, Modelling, and Projections (www.epidem.org) for substantial input and feedback throughout the development of this research. We thank Rich Fitzjohn for research software and computing support.

### Conflicts of interest

There are no conflicts of interest.

## Supplementary Material

Supplemental Digital Content
