# Supplementary material for: The Estimation and Projection Package Age-Sex Model and the r-hybrid model: new tools for estimating HIV incidence trends in sub-Saharan Africa
Source: AIDS. 2019 Nov 12;33(Suppl 3):S235–44. doi: 10.1097/QAD.0000000000002437 (PMC6919231; doi:10.1097/QAD.0000000000002437)
Supplement: Supplemental Digital Content [file aids-33-s235-s001.pdf]

Supplementary Information — EPP-ASM and the r-hybrid model: new tools for estimating HIV incidence trends in sub-Saharan Africa

Jeffrey W Eaton, Tim Brown, Robert Puckett, Robert Glaubius, Kennedy Mutai, Le Bao, Joshua A Salomon, John Stover, Mary Mahy, Timothy B Hallett

Contents

S1. Technical details of the EPP-ASM model 2

S2. Technical details of the random walk component of the r-hybrid model 10

S3. EPP-ASM likelihood specification 16

References 18

Figures and Tables

Figure S1: Diagramme of mixed stratification structure for the EPP-ASM model. 4

Figure S2: Sex and age stratified trends in prevalence, incidence, and AIDS mortality in Malawi Central Region estimated from EPP-ASM r-hybrid model. 8

Figure S3: Estimates of prevalence, incidence rate and AIDS deaths by single-year of age in 1995, 2005, and 2015 for Malawi Central Region from EPP-ASM r-hybrid model. 9

Figure S4: Outputs from the r-hybrid model using random walk knot spacings every 5, 3, and 1 years. 12

Figure S5: Outputs from the r-hybrid model using random walk knot spacings every 5, 3, and 1 years. 13

Figure S6: Posterior mean estimates for 177 EPP regions when using 5-year knot spacing or annual knot spacing. 14

Figure S7: Posterior uncertainty estimates for 177 EPP regions when using 5-year knot spacing or annual knot spacing. 15

Table S1: Number of IMIS iterations to achieve posterior convergence at varying random-walk knot spacings. 15

S1. Technical details of the EPP-ASM model

The role of the EPP model in constructing national HIV estimates and projections is to estimate the adult HIV incidence trend from available HIV survey and surveillance data. The estimated HIV incidence trend is provided to the Spectrum model, which uses the HIV incidence trend to calculate HIV epidemic and impact indicators such as PLHIV, HIV prevalence, antiretroviral treatment coverage, AIDS deaths, mother-to-child HIV transmission, paediatric HIV outcomes, and AIDS orphanhood. Ensuring consistency of outputs from the Spectrum with the data to which EPP is calibrated requires consistent model structure and assumptions that dictate relationships between HIV incidence, prevalence, and AIDS mortality by sex and age. To achieve this, the EPP-ASM model represents the adult population aged 15 years and older by sex, single-year of age, and HIV status, and mirrors the model structure and assumptions of the Spectrum model:

- The adult population is stratified by single-year of age and sex between ages 15 to 79 and 80+.
- The HIV population is stratified according to seven CD4 count stages: ≥500, 350-499, 250-349, 200-249, 100-199, 50-99, <50.
- The population on antiretroviral treatment stratified by CD4 stage at treatment initiation, and three treatment duration groups: 0-5 months, 6-11 months, and 12+ months.

A key design principle for EPP-ASM was to balance precise representation of the demographic and epidemiologic processes with computational efficiency to enable hundreds of thousands of model simulations during Bayesian model calibration. Representing the full stratification of the Spectrum model (single-year age groups, two sexes, eight stages of infection, and four treatment stages) and simulating transitions between all of these states with 0.1-year time step was not computationally practical. We also found that an alternative

approach of approximating ageing as a Markov transition through 5-year age groups did not provide sufficiently accurate representation of ageing.

To address this challenge, we developed a mixed approach in which populations are tracked with different levels of stratification and model processes simulated on different time steps (Figure S1). The total population is stratified by single-year of age, sex, and HIV status (HIV-negative and HIV-positive). Then amongst the HIV population, the stratification across CD4 stages and durations on ART are tracked only by nine coarser age groups 15–16, 17–19, 20–24, …, 45–49, 50+. HIV natural history processes—disease progression through CD4 stages, AIDS mortality from untreated and treated CD4 stages, and ART initiation—are simulated every 0.1-year time-step. The collapsing from 66 age groups to 9 coarser age groups substantially reduces the size of the state-space and hence the computational load for each 0.1-year time-step.

This structure implicitly assumes a homogenous distribution of the HIV population by CD4 category and ART duration within the coarser age group, while the exact size of the total HIV-positive population is tracked by single-age year. We found this provided very accurate representation of the Spectrum model, with the addition of the stratification of the 15–19 age group into 15–16 and 17–19 because HIV incidence changes rapidly within this age range, resulting in a rapidly changing CD4 distribution amongst those HIV-positive.

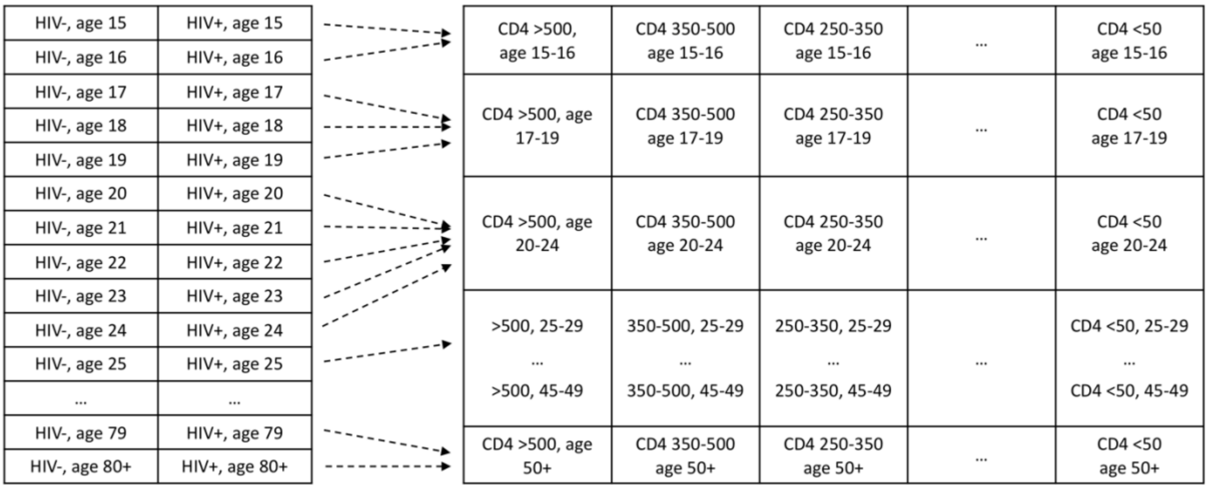

Figure S1: Diagramme of mixed stratification structure for the EPP-ASM model. The size of the HIV negative and HIV positive population is tracked by sex and single year of age from age 15 to 80+ (2 sexes x 66 ages x 2 HIV status; left). Within the HIV positive population, the stratification by CD4 stage and ART duration is tracked only by nine coarser age groups 15-16, 17-19, 20-24, 25-29, 30-34, 35-39, 40-44, 45-49, and 50+ (2 sexes x 9 age groups x 7 CD4 stages x 4 ART durations; right). Demographic processes (ageing, births, non-HIV mortality, migration) are simulated on an annual time step by single-year of age (mid-year to mid-year). HIV incidence is calculated and added to the HIV+ population by single-year of age with 0.1 year time step. Disease progression, AIDS mortality, and ART initiation are calculated within the coarser nine age groups on with 0.1 year time step. (Note: stratification by ART duration category is not illustrated in the diagramme.)

Demographic projection

Consistent with Spectrum, demographic projection of the HIV negative and HIV positive population occurs by single-year of age with annual time step from mid-year to mid-year. The following demographic input parameters are taken from Spectrum as annual inputs:

- Initial population size by age and sex in the projection start year, typically 1970;
- Probability of survival (S_x) from age x to age $x+1$ from causes of death other than HIV;
- Age-specific fertility rate;
- Male-to-female sex ratio at birth;
- Number of net migrants by age and sex *or* the 'target' mid-year population size by age and sex in each year.

If a target population is specified rather than the number of net migrants, at the end of each annual projection step (after simulating HIV processes), the population is scaled in each age/sex compartment to match the target population. This option is used as default for EPP regional stratifications (urban/rural or other subnational) in which demographic inputs and migration are challenging to estimate, but population size and distribution are available.
Since the model represents only the population aged 15 and older, the number of births by sex is calculated each year and stored as a lagged input to the model 15 years later, reduced by the calculated probability of survival from birth to age 15 for each cohort.
Modelling paediatric HIV, including mother-to-child HIV transmission, paediatric HIV progression and survival, and effects of paediatric ART on survival incurs substantial model complexity, but only modestly affects adult HIV inference. Rather than fully simulating these processes, the model uses the HIV prevalence among age 15 entrants and their distribution across CD4 and ART stages as fixed model inputs from a previous Spectrum simulation.

HIV incidence by age and sex

New HIV infections are calculated at every 0.1 year time step by sex and single year of age. The first step to calculating new infections is calculate the HIV incidence rate among adults age 15 to 49 years as a function of the transmission rate $r(t)$, HIV prevalence $\rho_{15\text{-}49}(t)$, and ART coverage $\alpha_{15\text{-}49}(t)$:

$$\lambda_{15\text{-}49}(t) = r(t) \cdot \rho_{15\text{-}49}(t) \cdot \left(1 - \omega \cdot \alpha_{15\text{-}49}(t)\right).$$

After calculating the HIV incidence rate in each time-step, the number of new adult infections are allocated by sex and single-year of age. The female-to-male incidence rate ratio and age-specific incidence rate ratio relative to age 25–29 years by sex in each year are taken as fixed inputs from Spectrum for ages 15-19 through 75-79, with no new infections assumed in the 80+ age group. Incidence rate ratios by five-year age group are disaggregated to single-year using Beer’s graduation coefficients [1], following Spectrum.

HIV disease progression, AIDS mortality, and antiretroviral treatment

The EPP-ASM model takes the following inputs from Spectrum for modelling HIV natural history and the impacts of ART on survival:

- Initial CD4 distribution following seroconversion by age and sex;
- Annual rate of progression to next CD4 stage by age and sex;
- AIDS mortality rate by CD4 stage, age, and sex;
- AIDS mortality rate for those on ART by age, sex, CD4 at ART initiation, treatment duration, and calendar year;
- Number or percentage of ART eligible adults on treatment at the end of each calendar year.
- CD4 threshold for ART eligibility in each calendar year;
- Percentage eligible for treatment due to other eligibility criteria (e.g. serodiscordant couples, TB infection, key population groups) by sex and year;
- optionally, the ART drop-out rate; and
- optionally, the median CD4 count at ART initiation in years for which it is known.

Disease progression, AIDS deaths, and ART initiations are calculated every 0.1 year timestep within the nine coarse age groups. At the end of this calculation, the number of AIDS deaths to the HIV positive population must be removed from the HIV positive population by single year of age (Figure S1; left) such that the total HIV positive population remains exactly aligned. Deaths are removed proportionally from the single-year age groups within each coarse age group proportionally to the distribution of the single-age HIV positive population within each coarse age group.

Example age-stratified outputs

Figure S2 and Figure S3 illustrate examples of sex and age stratified outputs from the EPP-ASM model applied to Malawi Central Region (shown in the second row of Figure 1). Figure S2 shows time trends in HIV prevalence, HIV incidence rate, and AIDS mortality rate by age

groups 15-24, 25-34, 35-49, and 50+ years. This illustrates the characteristic diverging trends in HIV prevalence by age of declining HIV prevalence amongst the young adults as incidence declines while it increases rapidly amongst older adults due to longer survival following ART scale up. Figure S3 shows estimates of HIV prevalence, HIV incidence rate, and AIDS mortality rate by single year of age for ages 15 to 64 for the year 1995, 2005, and 2015, illustrating the older age profile of HIV among men compared to women and again the increasing age of peak HIV prevalence as the epidemic matures.

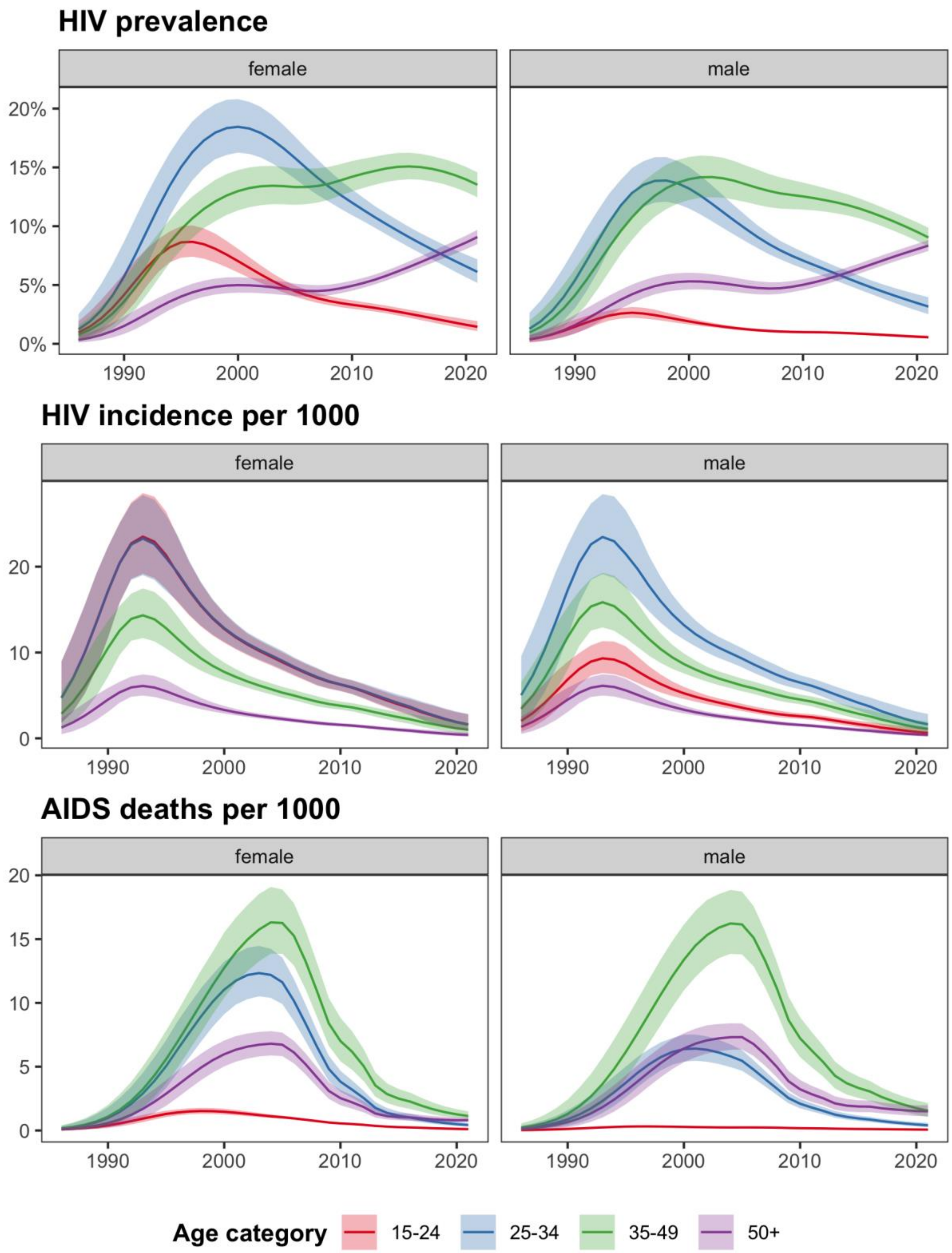

Figure S2: Sex and age stratified trends in prevalence, incidence, and AIDS mortality in Malawi Central Region estimated from EPP-ASM r-hybrid model. Lines illustrate posterior mean and shaded areas reflect 95% credible ranges.

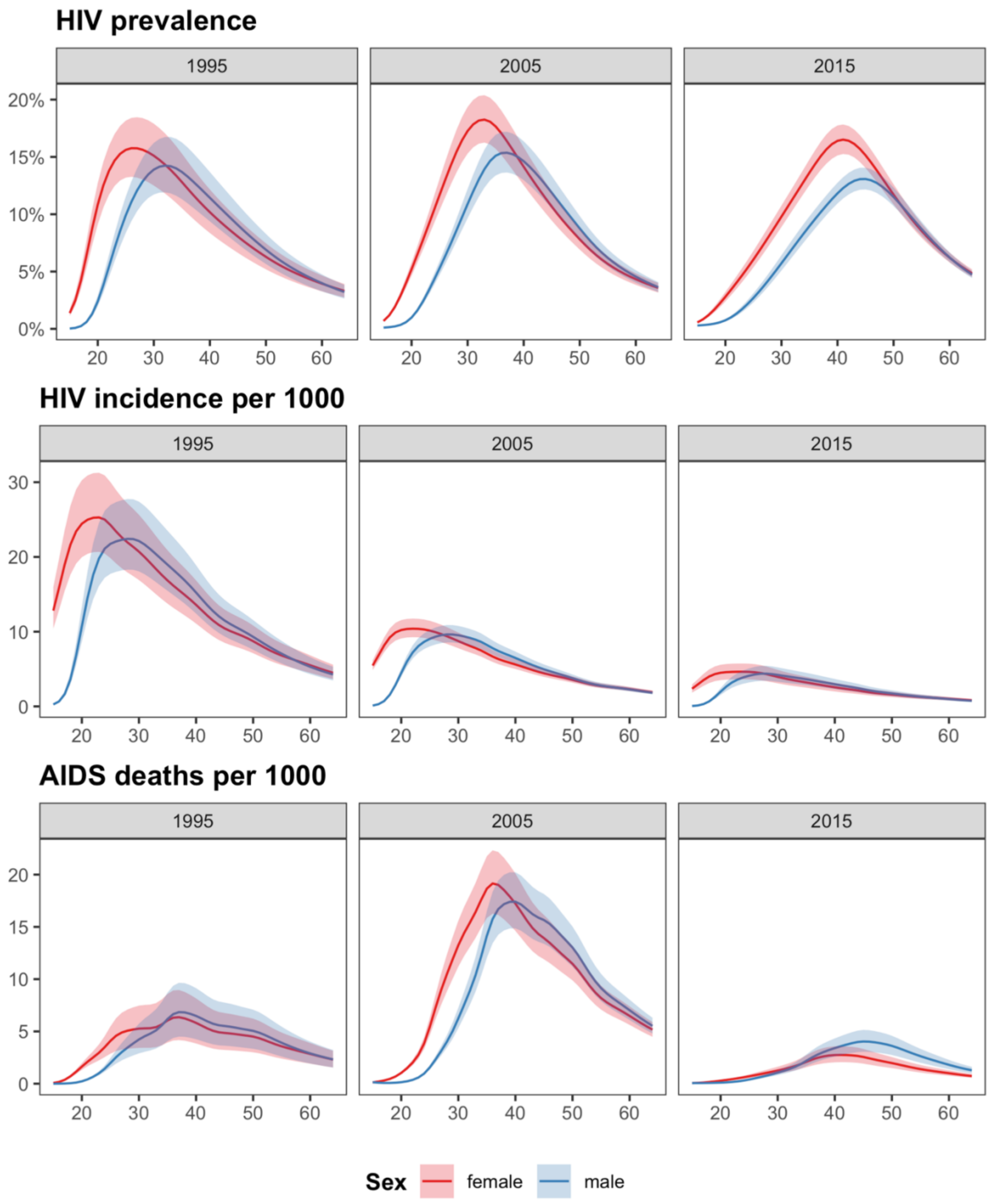

Figure S3: Estimates of prevalence, incidence rate and AIDS deaths by single-year of age in 1995, 2005, and 2015 for Malawi Central Region from EPP-ASM r-hybrid model.

S2. Technical details of the random walk component of the r-hybrid model

From the mid-2000s, the r-hybrid model uses a piecewise-linear spline with a first-order random-walk (RW1) penalty on the spline coefficients to model changes in $\log r(t)$.

The piecewise-linear spline is defined on a sequence of knots $\tau_0, \dots, \tau_K$ evenly spaced $\tau_i - \tau_{i-1} = \Delta_\tau$ years apart with τ_0 being the minimum time for the spline model and τ_K spanning the end of the model simulation. Then

$$\log r_{RW}(t) = \sum_{i=1}^{K} \beta_i \cdot \sqrt{\Delta_\tau} \cdot b_i\,(t)$$

where

$$b_i(t) = \begin{cases} 0, & t < \tau_{i-1} \\ t - \tau_{i-1}, & \tau_{i-1} < t \leq \tau_i \\ 1, & t \geq \tau_i \end{cases}$$

and the coefficients $\beta_i \sim Normal(0, \sigma_{RW})$. The standard deviation for the random walk is fixed at a default parameter value of $\sigma_{RW} = 0.06$. The term $\sqrt{\Delta_\tau}$ is included so that the dispersion of the random walk over time is invariant to the choice of knot spacing Δ_τ. To see this, observe that for any $j \in \{1, \dots, K\}$,

$$Var\left(\log r_{RW}(\tau_j)\right) = Var\left(\sqrt{\Delta_\tau} \cdot \sum_{i=1}^{j} \beta_i\right) = \Delta_\tau \sum_{i=1}^{j} Var(\beta_i) = j \cdot \Delta_\tau \cdot \sigma_{RW}^2 = \left(\tau_j - \tau_0\right) \cdot \sigma_{RW}^2.$$

As default, the random-walk model is initiated at $\tau_0 = 2003$ with a phased introduction over the five-year period 2003 to 2008 to ensure a smooth transition between the logistic model and random walk models:

$$\frac{d}{dt}\log r(t) = \begin{cases} \frac{d}{dt}\log r_{logis}(t), & t < 2003 \\ (1 - w(t)) \cdot \frac{d}{dt}\log r_{logis}(t) + w(t) \cdot \frac{d}{dt}\log r_{rw}(t), & t \in [2003, 2008) \\ \frac{d}{dt}\log r_{rw}(t), & t \geq 2008 \end{cases}$$

where the weighting function $w(t) = \frac{t-2003}{5}$ linearly interpolates between the two specifications over the period 2003 to 2008. For simulating this function as part of an EPP model projection, the differential equation is discretised and solved numerically via Euler integration at each 0.1 year time step required for EPP epidemic simulation.

We found that knot spacings every $\Delta_\tau = \{1, 2, 3, \text{and } 5\}$ years made negligible difference to posterior estimates, projections, and uncertainty ranges for HIV transmission rate, incidence, or prevalence, but that longer knot spacings substantially improved parameter identifiability and model convergence. Figure S4 illustrates results of the r-hybrid model fitted with $\Delta_\tau = 1$, 3, and 5 years fitted to the four EPP regions shown in Figure 1. The posterior mean and 95% CI ranges are nearly indistinguishable for the different knot spacing choices. These results are summarized for all 177 EPP regions in Figure S5 showing posterior mean estimates and Figure S6 showing posterior standard deviation. Figure S5 shows that posterior mean estimates are virtually identical when using annual knot spacing versus knots every 5 years. In Figure S6, the posterior uncertainty is only very slightly larger when using annual knots compared to knots every 5 years. Figure S7 and Table S1 summarise the median number of iterations required to achieve convergence of the IMIS algorithm. This steadily declines with the knot space, with the most dramatic reduction from $\Delta_\tau = 1$ to $\Delta_\tau = 2$. With $\Delta_\tau = 2$ to $\Delta_\tau = 5$, the number of iterations was lower than required for convergence of the r-spline model, and comparable to the r-trend model.

Based on these results, we recommended the default $\Delta_\tau = 5$, requiring $K = 5$ parameters for a spline spanning a projection from 2003 through 2025. In total, the r-spline model requires eight parameters (seven spline coefficients and one variance parameter) to specify $r(t)$ compared to nine for the r-hybrid model (four logistic function parameters and five random walk coefficients). The current EPP implementation allows different knot spacing to be

specified, and denser knot spacing may be reconsidered in future should sufficiently precise data become available to identify more frequent fluctuations in the HIV transmission rate.

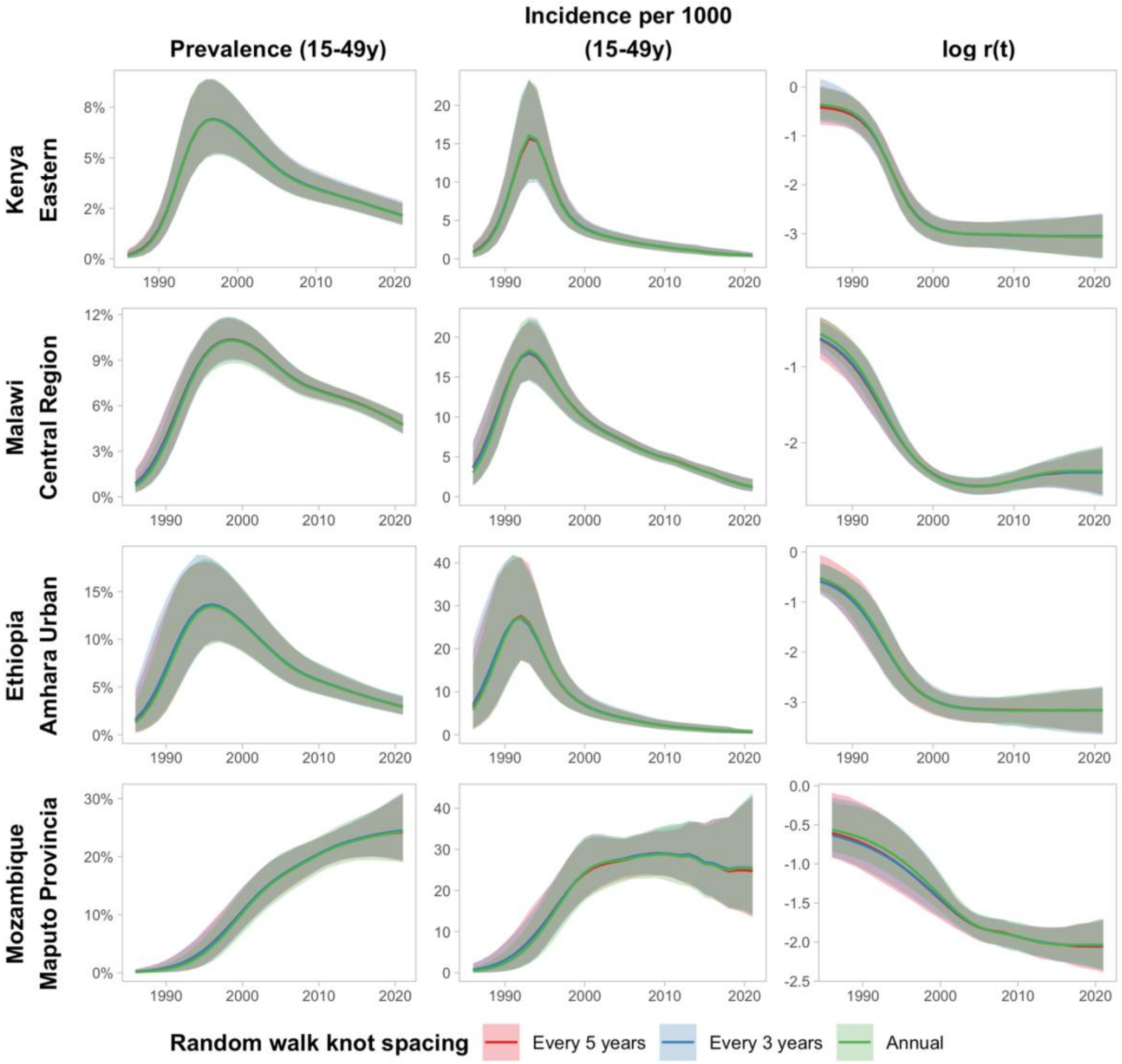

Figure S4: Outputs from the r-hybrid model using random walk knot spacings Δ_τ every 5, 3, and 1 years. Results show estimates and 95% credible intervals for trends in HIV prevalence (left), HIV incidence rate per 1000 (center), and log r(t) (right) for the same example regions presented in Figure 1. Estimates and uncertainty range bounds are nearly indistinguishable indicating that results are insensitive to knot spacings ranging from annual to every 5 years.

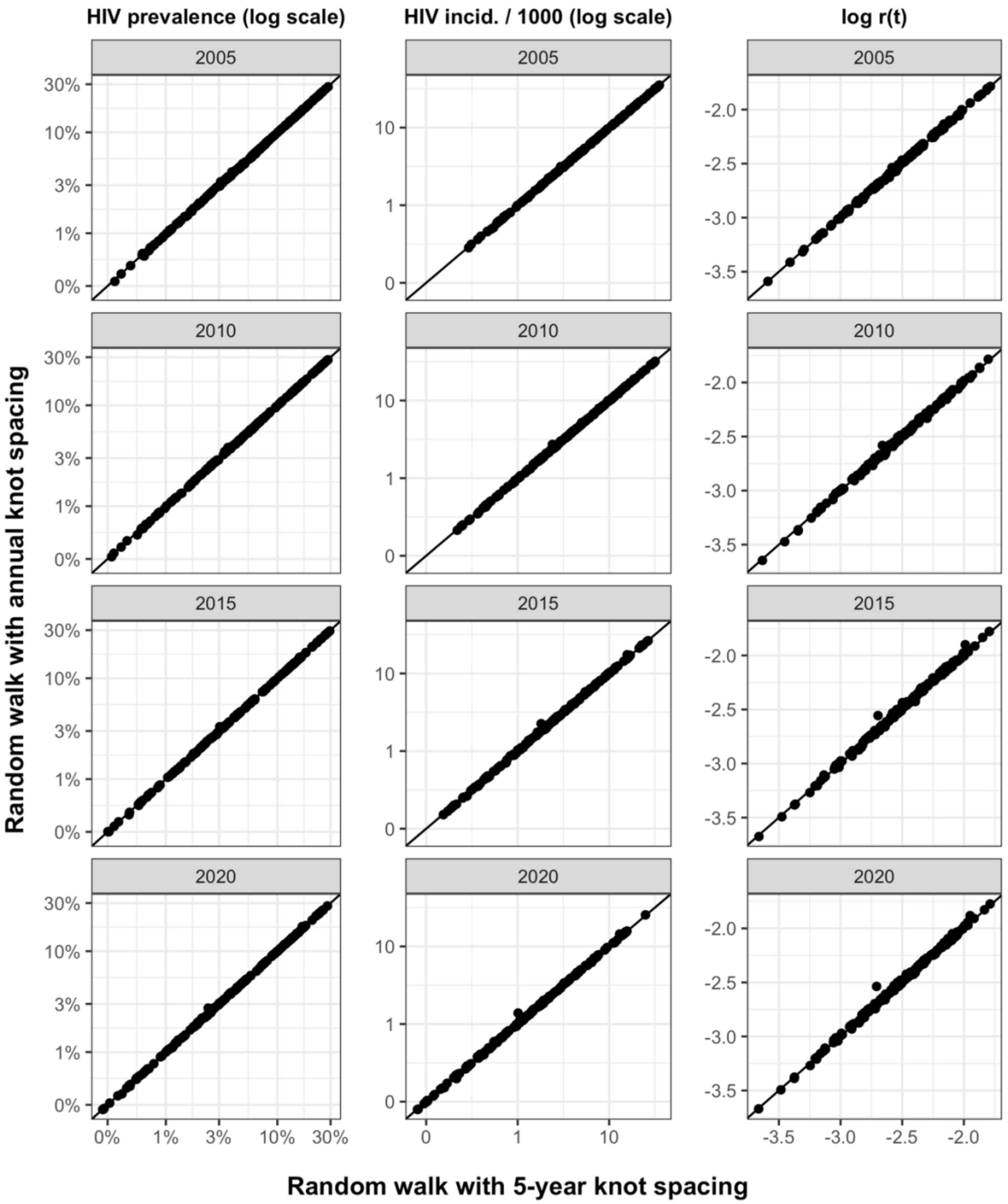

Figure S5: Posterior mean estimates for HIV prevalence (left), HIV incidence rate per 1000 (center), and $log\ r(t)$ (right) when using 5-year knot spacing ($\Delta_\tau = 5$) or annual knot spacing ($\Delta_\tau = 1$)for model fits to 177 EPP regions. Estimates are presented for years 2005, 2010, 2015, and 2020. The transition from logistic function to random walk begins in year 2003.

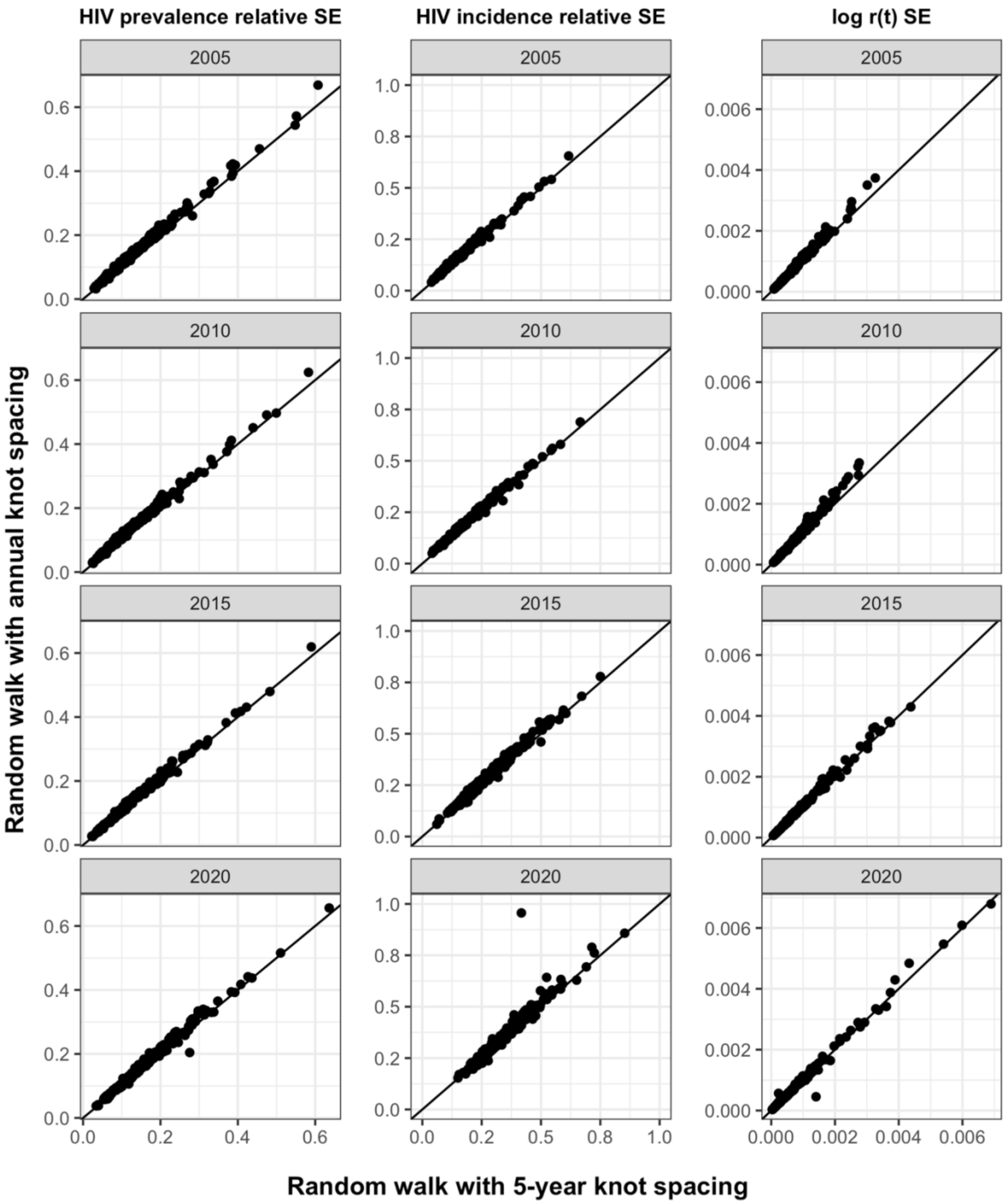

Figure S6: Posterior uncertainty estimates for HIV prevalence (left), HIV incidence rate per 1000 (center), and $log\ r(t)$ (right) when using 5-year knot spacing ($\Delta_\tau = 5$) or annual knot spacing ($\Delta_\tau = 1$) for model fits to 177 EPP regions. Estimates are presented for years 2005, 2010, 2015, and 2020. The transition from logistic function to random walk begins in year 2003. For HIV prevalence and HIV incidence rate, the relative standard error (standard error divided by mean) are plotted.

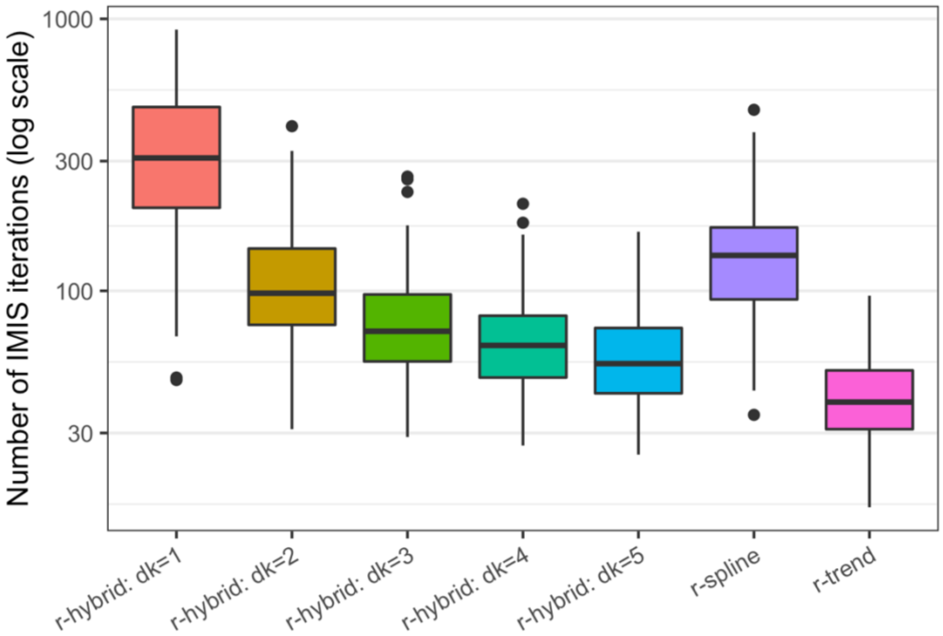

Figure S7: Number of IMIS iterations to achieve posterior convergence for random-walk knot spacings ranging from annual (dk=1) to every 5 years (dk =5) for model fits to 177 EPP regions. For comparison, the number of IMIS iterations for convergence of the r-spline and r-trend models are presented. All model fits used

Table S1: Number of IMIS iterations to achieve posterior convergence at varying random-walk knot spacings**.**

**Model**	**IMIS iterations**	
	Median	Interquartile range
r-hybrid: $\Delta_\tau = 1$	308	[202, 474]
r-hybrid: $\Delta_\tau = 2$	98	[75, 143]
r-hybrid: $\Delta_\tau = 3$	71	[55, 97]
r-hybrid: $\Delta_\tau = 4$	63	[48, 81]
r-hybrid: $\Delta_\tau = 5$	54	[42, 73]
r-spline	135	[93, 171]
r-trend	39	[31, 51]

S3. Likelihood details

Data about HIV prevalence population surveys in region x in year t are summarized as the observed HIV prevalence $\hat{p}_{x,t}$ with associated design-based standard error estimate $\hat{v}_{x,t}$. A normal distribution is used to approximate the likelihood for probit-transformed survey prevalence

$$\Phi^{-1}\left(\hat{p}_{x,t}\right) \sim Normal(\Phi^{-1}(\rho_{x,t}(\theta)), \tilde{v}_{x,t})$$

where the mean is the probit-transformed $\rho_{x,t}(\theta)$ model prediction for HIV prevalence as a function of the vector or parameters θ relevant a given EPP model choice for and the standard deviation $\tilde{v}_{x,t}$ is derived via the delta-method approximation

$$\tilde{v}_{x,t} = \sqrt{2\pi} \cdot \exp\left(0.5 \cdot \Phi^{-1}\left(\hat{p}_{x,t}\right)^2\right) \cdot \hat{v}_{x,t}.$$

For instances in which both HIV prevalence and incidence were measured in the same survey, we updated the previously described statistical model [2] with a new likelihood approximation that accounts for uncertainty about test for recent infection characteristics (mean duration of recent infection and false recent ratio) and complex survey design, and allows the user to input the final incidence estimate and standard error, rather than full details of the incidence rate calculation. Let $\{\hat{p}_{x,t}, \hat{h}_{x,t}\}$ be the estimated prevalence and incidence rate, respectively, with covariance matrix

$$\hat{V}_{x,t} = \begin{bmatrix} \hat{v}_{\rho}^{\wedge 2} & \hat{v}_{\rho,\lambda} \\ \hat{v}_{\rho,\lambda} & \hat{v}_{\lambda}^{\wedge 2} \end{bmatrix}$$

where $\hat{v}_{\rho,\lambda}$ is the covariance of the HIV prevalence and incidence estimates arising because the formula for estimating incidence $\hat{h}_{x,t}$ from recent infection status depends on the prevalence $\hat{p}_{x,t}$ [3] and clustered survey design. We modelled the observed probit-transformed prevalence and log-transformed incidence as a bivariate normal distribution

$$\begin{Bmatrix} \Phi^{-1}\left(\hat{p}_{x,t}\right) \\ \log \hat{h}_{x,t} \end{Bmatrix} = Normal_2\left(\begin{bmatrix} \Phi^{-1}(\rho_{x,t}(\theta)) \\ \log \lambda_{x,t}(\theta) \end{bmatrix}, \tilde{V}_{x,t}\right)$$

where $\tilde{V}_{x,t}$ is approximated via the delta method as

$$\tilde{V}_{x,t} = \begin{bmatrix} 2\pi \cdot \exp\left(\Phi^{-1}\left(\hat{p}_{x,t}\right)^2\right) \cdot \hat{v}_{\rho}^{\wedge 2} & \dfrac{\sqrt{2\pi} \cdot \exp\left(0.5 \cdot \Phi^{-1}\left(\hat{p}_{x,t}\right)^2\right)}{\hat{h}_{x,t}} \cdot \hat{v}_{\rho,\lambda} \\ \dfrac{\sqrt{2\pi} \cdot \exp\left(0.5 \cdot \Phi^{-1}\left(\hat{p}_{x,t}\right)^2\right)}{\hat{h}_{x,t}} \cdot \hat{v}_{\rho,\lambda} & \dfrac{1}{\hat{h}_{x,t}^2} \cdot \hat{v}_{\lambda}^{\wedge 2} \end{bmatrix}.$$

Data about HIV prevalence among pregnant women attending ANC may arise from sentinel surveillance conducted at sentinel sites $s \in \{1, \ldots, S\}$ in region x in years $t \in \{1, \ldots, t_s\}$ or as the aggregation of HIV testing of all pregnant women at all ANC facilities in region x, termed a 'census' of ANC routine testing (ANC-RT) data. The basic likelihood formulation for site-level ANC prevalence observations remains the site-level random effects model proposed by Alkema, Raftery, and Clark [4] with estimated additional non-sampling error variance described by Eaton and Bao [5]. The statistical models for ANC prevalence from ANC-RT at the site-level or census level are described by Sheng *et al.* [6]. The key difference in our formulation is that rather than modelling ANC prevalence as a function of general adult population prevalence $\rho_t^{15\text{-}49}(\theta)$, ANC prevalence is related to the HIV prevalence among pregnant women $\rho_t^{\text{preg}}(\theta)$ predicted by the EPP-ASM model accounting for age-specific fertility, age-specific HIV prevalence among women, and the relative fertility of HIV positive women by age, CD4 stage, and ART status.

References

1 Siegel JS, Swanson DA (David A, Shryock HS. *The methods and materials of demography*. Elsevier/Academic Press; 2004.

2 Bao L, Ye J, Hallett TB. Incorporating incidence information within the UNAIDS Estimation and Projection Package framework: a study based on simulated incidence assay data. *AIDS* 2014; **28 Suppl 4**:S515-22.

3 Kassanjee R, McWalter TA, Bärnighausen T, Welte A. A new general biomarker-based incidence estimator. *Epidemiology* 2012; **23**:721–8.

4 Alkema L, Raftery AE, Clark SJ. Probabilistic projections of HIV prevalence using Bayesian melding. *Ann Appl Stat* 2007; **1**:229–248.

5 Eaton JW, Bao L. Accounting for nonsampling error in estimates of HIV epidemic trends from antenatal clinic sentinel surveillance. *AIDS* 2017; **31 Suppl 1**:S61–S68.

6 Sheng B, Marsh K, Slavkovic AB, Gregson S, Eaton JW, Bao L. Statistical models for incorporating data from routine HIV testing of pregnant women at antenatal clinics into HIV/AIDS epidemic estimates. *AIDS* 2017; **31 Suppl 1**:S87–S94.
